# Effect of EDTA and silane on the bond strength of cementing agents of glass fiber posts to root dentin

**DOI:** 10.4317/jced.62838

**Published:** 2025-07-01

**Authors:** Renata de Freitas Campelo, José Evando da Silva-Filho, Ana Paula Caracas-de-Araújo, Mário Fernando de Góes, Vicente de Paulo Aragão Saboia, Polyanna Maria Rocha Novais, Eduardo Diogo Gurgel-Filho

**Affiliations:** 1 DDS, MsD. Private Dental Clinical Practice, Fortaleza, Ceará, Brazil; 2 DDS, MsD Student. Departments of Dental Radiology and Imaging and Endodontics, Faculty of Dentistry, University of Fortaleza, Fortaleza, Ceará, Brazil; 3 DDS, MsD Student. Department of Operative Dentistry, Faculty of Dentistry, University of Fortaleza, Fortaleza, Ceará, Brazil; 4 DDS, MSc, PhD, Department of Restorative Dentistry, Piracicaba Dental School, University of Campinas, Piracicaba, São Paulo, Brazil; 5 DDS, MsD, PhD. Department of Operative Dentistry, Faculty of Dentistry, Federal University of Ceará, Fortaleza, Ceará, Brazil; 6 DDS, MsD, PhD. Private Dental Clinical Practice, Fortaleza, Ceará, Brazil; 7 DDS, MsD, PhD. Department of Endodontics, Faculty of Dentistry, University of Fortaleza, Fortaleza, Ceará, Brazil

## Abstract

**Background:**

This study aimed to evaluate the effect of dentin pre-treatment with EDTA and silanization of glass fiber posts on the bond strength of different resin cements to root dentin.

**Material and Methods:**

Fifty-six extracted single-rooted human teeth received endodontic treatment and were restored with White Post DC (FGM) 0.5 fiber posts. Samples were divided into four groups: (1) Relyx U200 without EDTA, (2) Relyx U200 with EDTA, (3) Relyx ARC without EDTA, and (4) Relyx ARC with EDTA. Each group had a silane-treated subgroup (n=7). Root canals were prepared with Peeso reamer No. 3, leaving 4 mm of filling. Posts were cleaned with absolute alcohol (1 min) and air-dried (30 s). Silane was applied to half the posts for 60 s, then air-dried for 30 s. Specimens were mounted on acrylic plates with low-melting adhesive and sectioned using an Isomet saw (200 rpm, constant irrigation) and extra-fine diamond disc. Three slices (~1.5 mm thick) were obtained per tooth (coronal, middle, apical thirds). Thickness was measured with a digital caliper. Slices were positioned cervical side down in a 3.5 mm support matrix. A force was applied at 0.5 mm/min using a rod with a 0.8 mm extra-fine tip. Data were analyzed using Kolmogorov-Smirnov and Student’s t-tests.

**Results:**

Relyx ARC showed higher bond strength than Relyx U200, regardless of silane or EDTA use. The combination of silane and EDTA did not significantly affect results (Relyx U200 *p*=0.402; Relyx ARC *p*=0.510). Adhesive failure at the cement/dentin interface occurred in 51.61% of all samples, regardless of technique.

**Conclusions:**

EDTA and/or silane did not influence the bond strength of fiber posts to root dentin.

** Key words:**Bond Strength, Edetic Acid, Fiber-posts, Endodontics.

## Introduction

The different types of dentin surface conditioning directly influence the bond strength of teeth that have been endodontically treated with glass fiber posts cemented into their root canals (1-3). Conditioning dentin with phosphoric acid promotes rapid and deep smear layer removal and exposes the collagen matrix. This action aids in the creation of the hybrid layer, but it also activates the matrix metalloproteinase enzymes (MMPs) present in the collagen matrix, whose excessive activation can create voids that cannot be completely filled by the adhesive system. The activation of these enzymes contributes to faster degradation of the hybrid layer, decreased bond strength, and premature failure of the adhesive system (4-5).

Self-adhesive cements have been developed to circumvent potential operative errors by eliminating the need for prior dentin conditioning and smear layer removal, thus simplifying their application and reducing clinical time (6). Three-step etch-and-rinse adhesives are considered the gold standard.

Self-etch adhesives contain acidic monomers that promote partial demineralization of the dentin while allowing the bonding agent to penetrate, leading to the formation of mechanical micro-retentions (7). They differ from the conventional adhesive systems that condition the dentin separately, the latter enabling the formation of a thicker hybrid layer and better resin retention, which directly influences the bond strength of glass fiber posts to dentin structure.

Ethylenediaminetetraacetic acid (EDTA) is an agent used in dentistry for decades and has been tested as an effective dentin conditioner and potential MMP inhibitor. Its chelating ability removes calcium ions (Ca²+) from collagen matrices and binds to zinc ions (Zn²+) at the catalytic site of these proteolytic enzymes (8).

Some strategies may also be used to improve the bond strength between glass fiber posts and the cementing agent, such as treatment with hydrofluoric acid, sandblasting, hydrogen peroxide, or silane application on the surface of the post (9). The first treatments alter the post’s surface topography, thereby increasing mechanical retention, while silane is a chemical compound whose function is to bond the organic part of the cement to the inorganic portion of the glass fiber post. Despite its practical application in a single, quick, and simple step, its effectiveness remains controversial (10).

The present study aimed to assess the effects of dentin surface conditioning with EDTA on the bond strength of two cementing agents with different adhesion mechanisms: Relyx U200 and Relyx ARC. It also aimed to evaluate the effects of silane treatment or its absence on the surface of glass fiber posts.

## Material and Methods

- Ethics Approval

This study was approved by the research ethics committee (Approval No. 2.790.468).

- Sampling

The study included a total of 56 extracted single-rooted human teeth with a single root canal and fully formed apex. Teeth with canal atresia or accentuated curvatures and roots that presented caries and/or fractures in any thirds were excluded from the study. The specimens were kept hydrated in individual acrylic tubes with distilled water at room temperature. The coronal portion of each tooth was sectioned perpendicular to the long axis of the tooth, at the cementoenamel junction, using Carborundum discs mounted in a straight piece at low rotation, leaving a minimum size of 15 mm in root length.

- Preparation of the root canal

The canals were scouted using #10 Kerr-type endodontic files of 21 mm for locating the apical foramen and determining the working length (WL) 1 mm below the apex from apical patency. The pre-enlargement of the canals was performed with #15, #20 and #25 Kerr files of 21mm in the WL. While changing instruments and during their use, the canals were irrigated and flooded with 1ml of 2.5% sodium hypochlorite irrigating solution and apical patency was performed with a #10 Kerr file of 21mm. After pre-enlarging the canals, R#25 and R#40 reciprocating files were used in the WL to determine the surgical diameter (SD). The canals were then flooded with ethylenediaminetetraacetic acid (EDTA) for 3 minutes, irrigated again with 1 ml of 2.5% sodium hypochlorite, initially dried with an endodontic sucker and finished with absorbent paper cones.

The root canal was filled using standardized cones according to R#40 SD with a single Gutta-percha cone being used. Apical sealing and seating of the cone on the canal walls were verified through visual and tactile tests. The cement used was an epoxy amine-based polymer paste-paste system (AH Plus Dentsply, Intl) at a 1:1 ratio. The cones were soaked in the cement and placed inside the canal, with movements made throughout its long axis to guarantee the perfect filling with the cement. The access cavity was then sealed with provisional restorative material (Coltosol, Vigodent S/A Ind., Rio de Janeiro, RJ, Brazil). After endodontic treatment, the specimens were stored in distilled water at room temperature for a maximum of 30 days.

The canals were de-obstructed using a Peeso drill number 3 at low rotation with contra-angle handpiece and a specific de-obstructing drill for the post system itself with a diameter equivalent to a 0.5 post (WhitePost DC FGM), resulting in a remnant of filling material of 4 mm. Cleaning of the de-obstructed portion was performed with an exploratory probe No. 47 and 2.5% sodium hypochlorite using a clinical microscope (Alliance, São Carlos, SP, Brazil) at 25X magnification for better intra-canal visualization and cleaning of the dentin walls.

After de-obstructing and cleaning the canal, the teeth were divided into four different groups, with the cementing agent of the post and the treatment of the internal surface of the canal varying as follows: Group (1) RelyX U200 without EDTA; group (2) RelyX U200 with EDTA; group (3) Relyx ARC without EDTA; group (4) Relyx ARC with EDTA.

- Preparation of Glass Fiber Posts

Glass fiber posts with a diameter of 0.5 (WhitePost DC FGM) were used in all groups. All posts were washed with absolute alcohol for 1 minute and dried with a light blast of air for 30 seconds. A thin layer of silane (Monobond N, Ivoclar Vivadent) was applied to half of the samples in each group for 60 seconds and evaporated with blasts of air for 30 seconds.

- Experimental Groups

All root canals were washed with 2.5% sodium hypochlorite and dried using an endodontic sucker and absorbent paper cones. The respective cements were applied inside the root canal with the aid of a #40 Lentulo drill at low rotation followed by adaptation of the glass fiber post, removal of excesses, and photopolymerization for 8 seconds on each of the 4 sides of the root with VALO (Ultradent, 1400mW/cm²). RelyX U200 and Relyx ARC cements were used in 4 groups (G) and 2 subgroups (a and b), each with varying silanization of the post surface, ( Table 1).

For better contact of the adhesive system with the walls of the root canal, a long cylindrical cavibrush micro-applicator (Cavibrush FGM) was used. After cementing the posts, the specimens were kept in individual acrylic tubes, soaked in distilled water at room temperature for 24 hours.  Table 2 presents a summary of the materials used.

- Preparation of the samples

The specimens were fixed with low melt adhesive on an acrylic plate and then fitted into the sample holder of the precision cutting machine (Isomet 1000), being then cut with an extra-fine diamond disc at 200 rpm and with constant irrigation after the first millimeter of the cervical. A total of 3 samples measuring approximately 1.5 mm in thickness were obtained per tooth, one for each root third (coronal, middle and apical). The thickness of the samples was measured using a digital caliper (Manual Steel Caliper 150mm/6” Mtx).

- Push-out bond strength test

The samples were positioned with the cervical portion facing downwards on the support matrix with a 3.5mm hole for their stabilization. A force of 0.5mm/min was applied by means of a rod attached to the machine (Microtensile OM100- ODEME) with an extra-fine tip of 0.8 mm. The tip touches the center of the sample, on the portion corresponding to the post. The push-out tests provided values for the bond strength in grams (g), which were transformed into Newton (N), with 1g = 0.009807N, and then converted into megapascals (Mpa) by dividing the force value obtained by the area of the sample, as calculated by the following formula: Force (Mpa) = Force (N)/Area(mm2), where Area = (πr1+πr2) x L, and “L” is calculated using the formula √ (r1 - r2) 2+ h2, where “π” is the constant 3.14, “r1” is the largest radius, “r2” is the smallest radius and “h” is the thickness of the specimen.

After the push out tests, the specimens were evaluated and photographed using a clinical microscope (Alliance, São Carlos, SP, Brazil) with 25x magnification to determine the failure pattern of the samples. Failures were classified as adhesive failure between cement and dentin (CD), adhesive failure between post and cement (PC), failure due to fracture of either the post, the cement or the specimen (F) and mixed failure.

- Statistical analysis

The data obtained were tabulated in an Excel spreadsheet and statistically analyzed using SPSS version 23. The two types of cements, U200 and ARC, were analyzed to check for normality and compare the means in the use of silane and EDTA. The Kolmogorov-Smirnov test and Student’s t test were used with a significance level at 5% (*p*<0.05).

## Results

**Figure 1:**
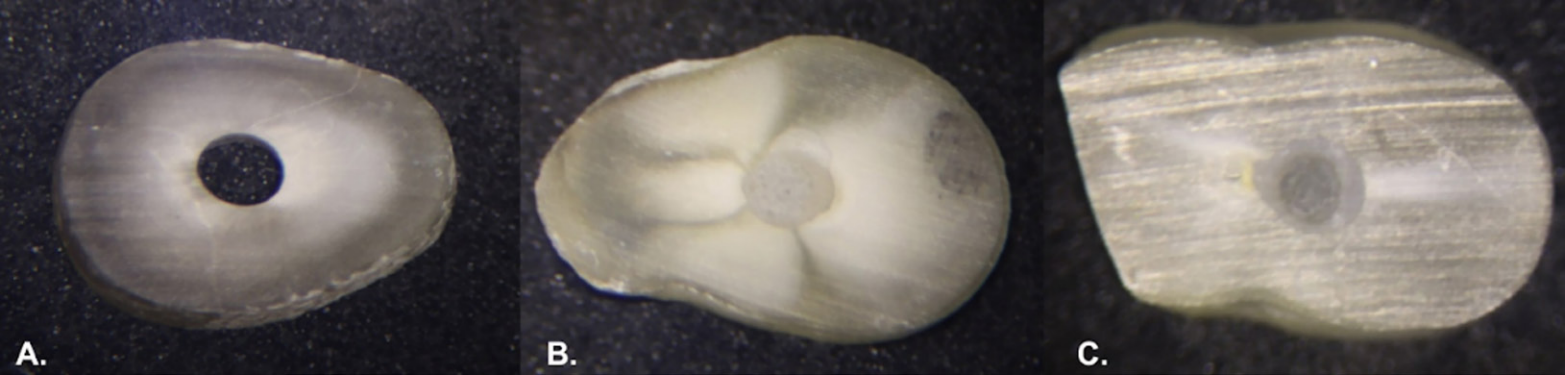
Adhesive failures under clinical microscope visualization at 25X magnification. A. Adhesive failure between cement and dentin (CD); B. Adhesive failure between post and cement (PC), C. Mixed adhesive failure (F).

The bond strength results for the U200 and ARC groups in the cervical third of the root showed no statistically significant difference regarding the use or non-use of silane on the fiberglass post surface and EDTA treatment on the root canal wall, as shown in  Table 3 and  Table 4. Adhesive failures were identified through clinical microscope visualization at 25x magnification, three distinct patterns were observed and illustrated in Figure 1.

In the middle third of the post, it was observed that, when using U200 cement, the application of silane with EDTA did not influence bond strength (*p*=0.016) compared to the group in which silane was not used. The use of EDTA did not increase the bond strength of U200 cement. The lowest bond strength was observed when U200 was used with silane. The best results were observed in the group with U200 used with both silane and EDTA, and also in the group without silane and without EDTA.

In the apical third of the root, in the U200 group, the use of EDTA had a positive influence on bond strength, with significantly higher values compared to the group that used silane without EDTA (*p*=0.002). The use of silane had no influence on bond strength. 

Different letters indicate significant differences (*p*<0.05) according to Dunn’s test for each cement. In the cervical third, no differences were observed between groups. In the middle third, Groups 2 and 5, as well as Groups 3 and 5, differed. In the apical third, Groups 2 and 3, Groups 2 and 5, and Groups 1 and 5 showed significant differences. In the middle and apical thirds of the posts in the ARC group, no influence of silane or EDTA on bond strength was observed, meaning no statistical differences were found between the different treatments, as shown in  Table 4.

Identical letters indicate no significant differences (*p*>0.05) according to Dunn’s test. For ARC cement, no significant differences were found between groups in any of the three thirds evaluated.

Cement-dentin bond failure occurred in 51.61% of the samples (217), regardless of the bonding technique used, being most prevalent in the U200 group. Post-cement bond failure was more prevalent in the ARC group, accounting for 22%. Cohesive failures involving fracture of the post, cement, or dentin occurred in only 15.66% (34/217) of the specimens.

## Discussion

Resin cements have become widely used due to their better mechanical and aesthetic properties compared to conventional cements and their ability to adhere to restorative material (11). The biggest problem related to fiber posts cemented with resin cements is the difficult adhesion process (12). Laboratory studies have shown that adhesion failure is due to the difficulty in hybridizing the root dentin (13).

Some studies assess adhesion in the different thirds of the root canal (14), given the clinical importance of adhesion to the three thirds. The three parts are equally important for adhesion to the whole, so the present study assessed data from the three thirds.

Many tests can be used to measure bond strength of glass fiber posts to root dentin, such as tensile, shear, pull-out, and push-out tests. The latter, which was used in the present study, is thought to provide more accurate information on bond strength values since the shear force occurs parallel to the dentin-adhesive interface (15).

EDTA may be a viable alternative to phosphoric acid, as it does not cause significant changes to the structure of collagen and generates less demineralization of the dentin, thus exposing a shallower depth of collagen fibers and producing a thinner hybrid layer when associated with an adhesive system (4). While the use of 40% phosphoric acid for 20 seconds demineralizes approximately 1.0 µm of dentin, EDTA at 0.5 mol/L used for 60 seconds creates a thin demineralization layer of approximately 0.4 µm (16,17).

According to Çalt and Serper (18), the use of EDTA as a dentin conditioning agent may vary in terms of concentration, combination, and application time. The researchers tested applications at two different durations – 1 minute and 10 minutes – using 17% EDTA associated with 5% sodium hypochlorite (NaOCl). They concluded that the 1-minute application associated with NaOCl is sufficient and efficient in removing the smear layer, whereas its use for 10 minutes causes excessive erosion of inter- and peritubular dentin and, therefore, should not be used. There is an important association between the product’s contact time and its action on the dentin surface. This is corroborated by Ramírez-Bommer *et al*. (19), who observed that the depth of dentin reaction to EDTA increased with exposure duration. Treatment with EDTA associated with NaOCl results in greater degradation of collagen compared to using either substance individually. Because of this, the present study used 17% EDTA associated with NaOCl for 1 minute.

The adhesion of self-adhesive cements, such as Relyx U200, seems to be associated with the presence of the smear layer, relying on chemical interaction between its acidic monomers and the calcium hydroxyapatite in root dentin, providing micromechanical retention (20,21). Therefore, the use of NaOCl and EDTA, which remove this layer and expose collagen thereby allowing better penetration of adhesive systems – does not favor this type of cement (22). This was also observed in the present study. Unlike what was observed in relation to the ARC cement used in this study, the bond strength of the post-cement-dentin complex did not benefit from EDTA treatment of the root canal. Thus, the null hypothesis that EDTA treatment inside the root canals does not influence adhesion to root dentin should be accepted.

Therefore, excessive removal of calcium hydroxyapatite is detrimental to the bond strength of self-adhesive resin cements to root dentin (23). In the present study, during the use of U200, the root dentin treated with 17% EDTA was not beneficial for bond strength values. The same result was observed by Barreto *et al*. (23). On the other hand, for apical thirds, EDTA improved the bond strength, which can be explained by post locking or the irregular anatomy of dentinal tubules with limited action of EDTA. According to Luque-Martinez *et al*. (24), EDTA conditioning improves self-etching adhesive restoration retention rates 18 months after the procedure.

One of the strategies to improve retention of glass fiber posts is the use of surface pre-cementation treatments such as sandblasting, hydrofluoric acid, phosphoric acid, alcohol, silane, and hydrogen peroxide (25-28). The literature is still controversial regarding the use of silanizing agents (27,29). Some studies claim that treating the surface of the glass fiber post with silane is effective in increasing bond strength (10,30), while others disagree (31), corroborating the results found in the present study, as shown in  Table 3 and  Table 4. The null hypothesis that silanization of the glass fiber post does not influence bond strength regardless of the cement used was accepted.

According to Maroulakos *et al*. (28), surface treatment of the post is not always effective. Silane can improve bond strength, but the fiber post must have functional groups that react with the agent. Ineffectiveness may be due to weak or no chemical interaction between the silane monomer and the post, which may have few or no silanizable fibers exposed (26). However, it can be effective if associated with other pre-treatments.

In the present study, the use of silane did not interfere with bond strength values for U200. This result was also observed by Maroulakos *et al*. (28), who stated that surface treatment is not always effective. Silane can improve bond strength, but only if the post has reactive functional groups. Its ineffectiveness may stem from weak or absent chemical interaction with the post or the presence of few silanizable fibers (26). However, silane may be effective when associated with other post surface treatments. Bond strength may also be influenced by differences in silane composition and drying temperature, as different solvent evaporation methods and excess non-reactive components can impact bond strength. Nonetheless, the ideal protocol remains uncertain.

Predominant bonding failures occur at the cement-dentin interface, a critical region (32). In the current study, adhesive failure between cement and dentin (CD) was predominant at 51.6%, as expected. Similar results (53.9%) were observed by Barreto *et al*. (23). Adhesive failure between cement and post (PC) was 22%, very similar to the results of Barreto *et al*. (23), who found 19.5%. Dentin cohesive (DC) failures accounted for 15.66% of specimens. Bonding to root canal dentin remains a challenge due to root anatomy, handling characteristics of adhesive systems, and technique sensitivity.

More studies are needed to assess the effectiveness of EDTA and silane on the bond strength of glass fiber post cementation. These should include long-term follow-ups, such as the study by Simões *et al*. (33), which found that bond strength increased in samples cemented with EDTA, silane, and U200 after 6 months.

Based on the findings of the present study, we can conclude that the use of silane and EDTA did not influence the bond strength of the posts to root dentin regardless of the cementing agent.

## Figures and Tables

**Table 1:** G1a-Cementation with Relyx U200; G1b-Silanized posts cemented with Relyx U200; G2a-EDTA used in the canal and posts cemented with Relyx U200; G2b-EDTA used in the canal and silanized posts cemented with Relyx U200; G3a- Cementation with Relyx ARC; G3b: Silanized posts cemented with Relyx ARC; G4a: EDTA used in the canal and posts cemented with Relyx ARC; G4b: EDTA used in the canal and silanized posts cemented with Relyx ARC.

	G1a	G1b	G2a	G2b	G3a	G3b	G4a	G4b
EDTA 1 minute			X	X			X	X
Silane 1 minute		X		X		X		X
Relyx U200	X	X	X	X				
Relyx ARC					X	X	X	X
35% phosphoric acid					X	X	X	X
3-step adhesive					X	X	X	X

**Table 2:** Materials, composition, and methods of application used in the study.

Material	Composition	Application
Ethylenediaminetetraacetic acid (EDTA) / Biodynamics.	17% Tetrasodium salt aqueous solution (1 M; pH 7.2).	Apply until the canal is flooded, stirring manually with a file. Let it act for 1 minute and then wash with 2.5% sodium hypochlorite. Remove excess with a sucker and dry with an absorbent paper cone.
Ultra Etch Indispense/Ultradent Phosphoric Acid Etchant.	35% Phosphoric Acid.	Apply for 15 seconds in the root canal and then wash with distilled water for 30 seconds. Remove excess with a sucker and dry with an absorbent paper cone.
Monobond N Universal Primer/ Ivoclar Vivadent	Alcohol solution of silane methacrylate, phosphoric acid methacrylate and sulphide methacrylate.	Apply for 1 minute and gently air dry.
RelyX U200 Automix / 3M ESPE	Paste A—amine (bis-GMA), (TEGDMA), photoinitiators, inorganic silica and zirconia particles, and pigments. Paste B—TEGDMA, bis-GMA, inorganic silica and zirconia particles, benzoyl peroxide.	Mix them in equal parts for 10 seconds and then put them into the canal with the aid of a lentulo drill at low rotation. Wait 5 minutes and polymerize.
RelyX ARC / 3M ESPE	Silane treated ceramic, TEGDMA, bisGMA, silane treated silica, functional dimethacrylate polymer.	Mix them in equal parts for 10 seconds and then put them into the canal with the aid of a lentulo drill at low rotation. Wait 5 minutes and polymerize.
ScotchBond Multi-Purpose Plus (SBMP) / 3M ESPE	SBMP Activator: ethyl sulfinic acid salt solution and photoinitiator; Primer: 2-hydroxyethyl methacrylate (HEMA) and polyalkenoic acid copolymer; SBMP catalyst: bis-GMA, HEMA and benzoyl peroxide.	Apply activator and dry gently for 5 seconds; Apply primer and dry gently for 5 seconds; Apply the Catalyst and remove the excess with an absorbent paper cone.
WhitePost DC/ FGM Glass Fiber Post	80% Glass Fiber 20% Epoxy Resin	Place it inside the root canal.

**Table 3:** Results for the U200 group in the three root thirds evaluated.

Cement	Group	Cervical Third			K-W Test
		Median	Minimum	Maximum
U200	G1: U200 w/o Silane and w/o EDTA	12,05 a	3,37	14,70	0,100
	G2: U200 with Silane and w/o EDTA	5,16 a	3,74	7,03	
	G3: U200 w/o Silane and with EDTA	5,65 a	2,73	9,59	
	G5: U200 with Silane and with EDTA	5,78 a	5,09	9,70	
	Group	Middle Third			
	G1: U200 w/o Silane and w/o EDTA	6,85 a,b	2,68	15,56	0,016
	G2: U200 with Silane and w/o EDTA	3,86 a	1,71	8,42	
	G3: U200 w/o Silane and with EDTA	4,74 a	3,23	8,09	
	G5: U200 with Silane and with EDTA	8,32 b	5,39	17,17	
	Group	Apical Third			
U200	G1: U200 w/o Silane and w/o EDTA	6,17 ac	2,59	7,93	0,002
	G2: U200 with Silane and w/o EDTA	3,03 a	2,07	6,39	
	G3: U200 w/o Silane and with EDTA	6,35 b,c	4,44	14,84	
	G5: U200 with Silane and with EDTA	10,49 b	6,24	16,91	

**Table 4:** Results for the ARC group in the three root thirds evaluated.

Cement	Group	Cervical Third			K-W Test
		Median	Minimum	Maximum
ARC	G4: ARC w/o Silane and w/o EDTA	11,64 a	6,07	19,26	0,252
	G6: ARC w/o Silane and with EDTA	15,65 a	9,21	17,55	
	G7: ARC with Silane and with EDTA	12,80 a	6,33	17,18	
	G8: ARC with Silane and w/o EDTA	10,29 a	4,98	15,29	
	Group	Middle Third			
ARC	G4: ARC w/o Silane and w/o EDTA	8,69 a	5,81	12,25	0,166
	G6: ARC w/o Silane and with EDTA	12,64 a	10,48	18,36	
	G7: ARC with Silane and with EDTA	9,52 a	4,44	19,54	
	G8: ARC with Silane and w/o EDTA	13,98 a	5,95	17,88	
	Group	Apical Third			
ARC	G4: ARC w/o Silane and w/o EDTA	9,88 a	5,17	11,70	0,466
	G6: ARC w/o Silane and with EDTA	12,04 a	8,04	15,17	
	G7: ARC with Silane and with EDTA	11,10 a	5,61	14,63	
	G8: ARC with Silane and w/o EDTA	10,56 a	6,10	15,74	

## Data Availability

The datasets used and/or analyzed during the current study are available from the corresponding author.
